# Have Deaths of Despair Risen during the COVID-19 Pandemic? A Systematic Review

**DOI:** 10.3390/ijerph191912835

**Published:** 2022-10-07

**Authors:** Hania Rahimi-Ardabili, Xiaoqi Feng, Phi-Yen Nguyen, Thomas Astell-Burt

**Affiliations:** 1 Centre for Health Informatics, Australian Institute of Health Innovation, Macquarie University, Sydney 2109, Australia; 2 Population Wellbeing and Environment Research Lab (PowerLab), Wollongong 2522, Australia; 3 School of Population Health, Faculty of Medicine and Health, University of New South Wales, Sydney 2052, Australia; 4 School of Health and Society, Faculty of Arts, Social Sciences and Humanities, University of Wollongong, Wollongong 2522, Australia; 5 School of Public Health and Preventive Medicine, Monash University, Melbourne 3800, Australia

**Keywords:** deaths of despair, overdose, suicide, COVID-19, systematic review

## Abstract

This systematic review synthesized literature on potential impacts of protracted isolation and other disruptions during the COVID-19 pandemic on deaths of despair (suicide, overdoses, and drug-related liver diseases). Five electronic databases were searched yielding 70 eligible articles. Extant evidence mostly from high-income countries indicates COVID-19-related disruption may not have influenced suicide rates so far, but there have been reports of increased drug-related and liver disease mortality. Minority groups and women were more vulnerable, indicating the need for stronger equity focus on pandemic recovery and resilience strategies. Further high-quality studies with longer-term follow-up, especially from low-income countries, will inform these strategies.

## 1. Introduction

Beyond the acutely devastating rise in communicable disease mortality, impacts of the protracted socioeconomic disruption unleashed by the COVID-19 pandemic on population health are still emerging [1,2,3,4,5]. Early reports include potential aggravation of depression and anxiety [1,2], increases in suicidal ideation and behaviour [3,4], and drug overdoses [5]. These preliminary findings align with epidemiological studies of previous economic downturns, such as the global financial crisis of 2008-9, which had dire consequences for population health and health equity [6,7]. While some health impacts may be concurrent with crisis (e.g., stress), others manifest over time as biopsychosocial risk factors such as job loss, food insecurity, precarious housing availability, death of a loved one, and exposure to violence accumulate and in some cases overcome individual resilience [8]. This quantum of social determinants commonly experienced during economic downturn can induce and aggravate a sense of despair (derived from ‘desperare’, meaning ‘down from hope’ [9]) that undermines individual and shared meaning-making [10].

Despair, often in concert with concomitant factors such as loneliness, is thought to have been aggravated by social isolation practices enacted to perturb the spread of COVID-19, may lead to future discounting of health-risk behaviours (e.g., alcoholism and substance misuse) and increased risks of death from drug-related poisoning, liver diseases and suicide [11]. Despair initially was considered a clinical construct such as core symptoms of depression. Further investigations show despair manifests not only in cognitions but also in emotions and behaviours [10]. 

Case and Deaton coined the phrase ‘deaths of despair’ to describe these causes of death, and first reported an increase in deaths of despair which includes deaths as a result of self-destructive health behaviours (e.g., alcoholism) and suicide for non-Hispanic middle-aged White people in the US in 2015 [11,12]. Their study emphasised the role of underlying economic factors such as declining incomes and social factors such as ethnic discrimination and social isolation on the concerning rise of deaths of despair in this group [11,12]. Since then, other studies have indicated similar increases in other ethnic groups and countries [11,12,13]. Based on prior evidence an increase in deaths of despair induced by the COVID-19 pandemic is highly plausible. However, while some work has reported deaths of despair rising in the US in 2020 above pre-COVID-19 levels [14], there remains no systematic review of the literature to determine if this is an isolated case, or whether it is reflective of wider trends. This systematic review aims to resolve this gap in knowledge.

## 2. Materials and Methods

This review was conducted according to the Preferred Reporting Items for Systematic Review and Meta-Analyses (PRISMA) guidelines for systematic reviews [15]. Study outcomes were defined based on Case and Deaton’s definition of death of despairs, i.e., suicide (ICD10 X60-84, Y87.0), poisonings (ICD10 X40-45, Y10-15, Y45, 47, 49), and alcoholic liver diseases and cirrhosis (ICD10 K70, K73-74). ‘Poisonings are accidental and intent-undetermined deaths from alcohol poisoning and overdoses of prescription and illegal drugs’ [11].

### 2.1. Study Selection

Articles were included if they evaluated the deaths of despair during the COVID-19 pandemic. Due to the sensitivity of the subject of COVID-19, studies that have been published as editorials and letters to expedite the publication process were included if they used original objectively collected data. Table 1 outlines detailed inclusion and exclusion criteria.

### 2.2. Search Strategies

The following electronic databases were searched on 29 August 2021: Medline, Embase, Scopus, CINAHL, and PsycINFO. The search strategy was partially adapted from previous systematic literature reviews [3,6]. The COVID-19 search strings were used when there was no related filter available within the database using strings developed by librarians [16,17]. We used term keyword combinations of ‘despair’ and ‘deaths’ and ‘COVID-19’ searched in titles and abstracts (Table S1).

Study selection was completed via a two-step screening process using Covidence software (Veritas Health Innovation, Melbourne, Australia). Two reviewers (HR-A and PN) independently screened title/abstracts then full texts to identify eligible articles. Any disagreements were resolved by reviewing full texts and by discussion among investigators. The reference lists of the relevant articles were also reviewed by one reviewer (HR-A) to identify any eligible studies missed in the initial search process.

### 2.3. Data Extraction

One author (HR-A) extracted and synthesised data from the included articles into an Excel sheet.

The extracted data included author information, year of publication, study area, study design, population, and sample size. In addition, we collected data on the period that the data were collected, methods used to measure outcomes, outcomes (with ICD 10 if reported), comparison period, statistical analysis, covariates adjusted, main results, and mediating and moderating factors if assessed. Comparison time periods were divided into two categories of (1) a period leading to the pandemic (e.g., January–February 2020 vs. March–April 2020) or (2) the same time period of the previous year(s) (e.g., March–May 2019 vs. March–May 2020). Countries were classified into two categories of low- to upper-middle-income and high-income countries based on the WHO definition [18]. The direction of changes in death outcomes between COVID-19 and pre-COVID-19 periods are presented as increased, decreased, or no change. The majority of studies defined their cut-point for pandemic according to the date/month that the state of emergency was declared or lockdown measures introduced.

### 2.4. Quality Assessment

The National Heart, Lung, and Blood Institute quality assessment tools were used to evaluate the qualities of ecological and cross-sectional included articles [19]. Three further items were added for ecological studies [20]. For case report and case series studies, critical appraisal tools developed by the Joanna Briggs Institute were used [21]. For each item in the list, three options for answers were suggested, which were ‘Yes’, ‘No’ or ‘Other’ (NR, NA). If the criteria were met (Yes), it was assigned to the value of 1, otherwise, 0 points were assigned. The scores below 50, between 50 and 74, or above 75 meant the articles were regarded as low, fair, and high quality, respectively. The same classification was also used previously [22]. One reviewer (HR-A) conducted the quality assessment.

## 3. Results

**Figure 1:**
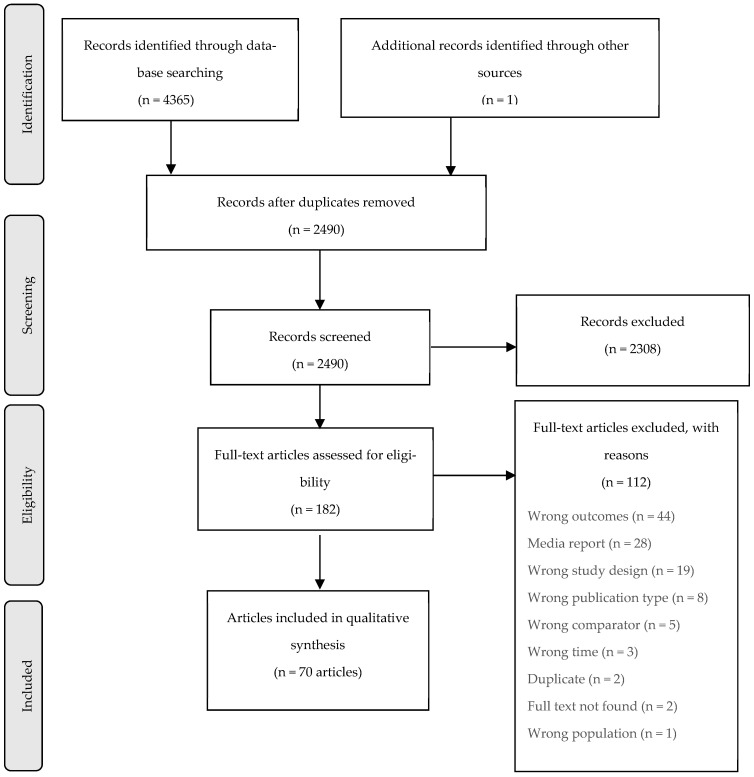
PRISMA flow diagram.

After removing duplicates, 2490 articles remained, 2308 articles did not meet the eligibility criteria and were excluded at the title/abstract screening stage. Full texts of 182 articles were reviewed, and 70 articles were selected for this systematic review (Figure 1). Three articles described two different outcomes, and one article [23] was an update of an earlier study [24]. Forty studies were on suicide deaths, 30 on overdose deaths, 2 on alcohol-related liver disease deaths, and 1 on hanging and poisoning (all intent) deaths. Studies were either funded by public organisations or had no funding.

### 3.1. Study Characteristics

Most of the articles (80%, *n* = 56) were published in 2021. Seventeen studies may not have gone through a peer-review process (e.g., editorial) [23,24,25,26,27,28,29,30,31,32,33,34,35,36,37,38,39,40]. Almost every study analysed objective data, except for one [31].

**Figure 2:**
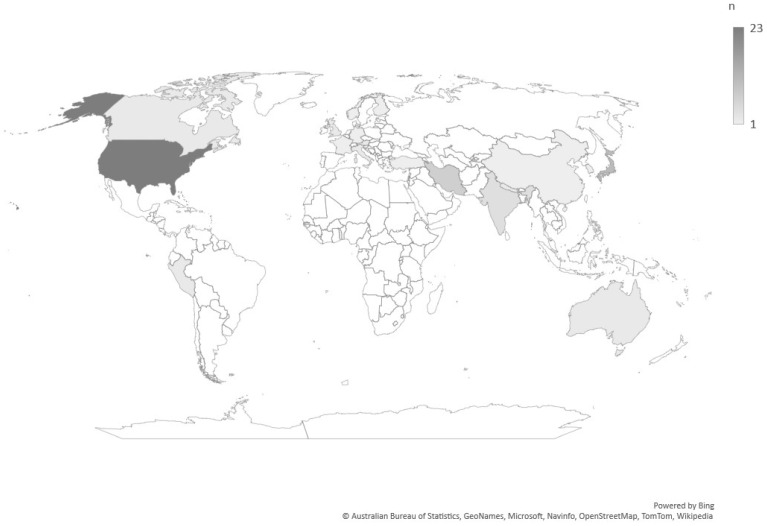
Geographical distribution of articles included. An article that considered 21 countries in their analysis is not considered in this figure.

Studies were mainly ecological or cross-sectional in design (*n* = 61). The COVID-19 study period varied from one month to one year. Overall, 17 countries were included. Some countries were studied several times, such as the US (*n* = 28) and Japan (*n* = 11), considering different states, populations, or periods. Figure 2 demonstrates the geographical variation in the studies included. Studies were mainly targeted the general population or adults. There was no consistent pattern in the time compared (i.e., period preceding COVID-19 or period at the same time of previous years). The study characteristics and findings are summarised in Tables S2 and S3 for suicide and drug-related deaths. Over half of the studies (*n* = 38) were judged to be of low quality, with only eight studies rated as high quality (Table S4).

### 3.2. Suicide Deaths

Of 40 studies that examined suicide, four were case reports/series [41,42,43,44], and 11 studies did not conduct any inferential analysis (testing hypotheses statistically) [30,31,32,33,37,38,45,46,47,48]. Findings are grouped based on country income and presented below.

All studies in developed countries found an overall no change (*n* = 4) or a declining trend (*n* = 10) except for studies conducted in Japan in the later months of the pandemic. Japan showed a declining trend for the first three months, but then suicide increased. In Italy, in the two months of February (beginning of pandemic) and April 2020 (the highest COVID-19 daily death), the suicide rate was higher than the rate for the same period in the previous year. Studies (*n* = 15) conducted in Japan [23,24,36,49,50,51,52,53], Australia [54,55], Austria [27,56], US [57], Canada [58], Germany [59], and the study that included in 21 countries [8] used adjustment for the time confounder (i.e., suicide trend) in their analysis.

Low- to upper-middle-income countries included in this review were India [25,42,48], Peru [60,61], Nepal [37,62], China [63], Sri Lanka [33], Turkey (case report) [41], and Iran (case reports) [43,44]. Inconsistent findings in two studies from different areas of India were reported. One study from New Delhi showed an initial decline with an increase in the post-lockdown period reaching the pre-COVID-19 rate [25]. The other study [48] reported similar findings in Nepal [37,62], with an overall increase in suicide deaths. The study findings from Peru [60,61], have a similar pattern as the New Delhi study; China reported an 18% decline [63]. A study in Sri Lanka assessed self-poisoning (intentional) death rate during COVID-19 compared with pre-pandemic rates and found a drop in numbers [33]. In addition to studies that examined suicide, one study descriptively assessed unnatural deaths over the 6 months of the pandemic at North Bengal in India and reported that persons involved in private jobs (44%) were more likely to commit suicide than those in the government jobs [64].

### 3.3. Overdose Deaths

Of 30 studies, 5 were case report/series or descriptive (i.e., no comparison), and 16 did not conduct any inferential analysis. Only three studies used considered adjusting for the time trend confounder [28,65,66]. Studies were conducted in six countries (US, UK, Canada, France, China, and Iran).

There were seven studies that assessed poisoning from ingestion of illicit alcohol (e.g., methanol poisoning). These studies were conducted in US (*n* = 1, case series) [39], France (*n* = 1, case report) [67], and Iran [35,68,69,70,71] (*n* = 5). However, the intention of ingestion was not evident in these studies. It is unclear whether the consumption of illicit alcohol is for recreational purposes or due to the spread of misinformation documented in some instances about disinfecting the digestive tract to prevent COVID-19 infection [68]. Thus, these studies were treated separately. Of these studies, five that compared deaths from methanol poisoning with pre-pandemic figures or previous methanol poisoning outbreaks all showed a considerable increase [35,68,69,70].

Most of the studies that conducted a comparison between pre- and post-pandemic periods (*n* = 20) showed that overdose death increased (*n* = 15 [28,29,34,40,65,66,72,73,74,75,76,77,78,79,80,81], 7 with significant findings [28,29,65,66,72,73,78]), compared with pre-pandemic figures. Only a few studies found null results (*n* = 3) [82,83,84] or a decrease in overdose death (*n* = 2) [63,85].

Five studies investigated the type of substance overdosed [72,73,79,81,82] or examined a specific drug [78,83]. Findings from these studies showed that the use of fentanyl (*n* = 4) [72,73,81,82], and stimulants such as cocaine and amphetamines [73,82] increased significantly compared with the pre-pandemic period. Further, of three studies measuring alcohol overdose deaths, all demonstrated an increase after the pandemic, with one showing the rise being considerably higher than other drug deaths (5.5-fold increase versus 2.5 fold) [79]. Findings on deaths from heroin, benzodiazepines, and fentanyl analogues overdose were inconsistent and limited [72,81,82]. Studies that examined overdose deaths related to prescription opioids [81,82] or overdose deaths among patients receiving treatment for substance misuse [83,84] did not observe any change during the pandemic.

### 3.4. Liver Disease

Two studies assessed alcohol-related liver disease or cirrhosis deaths, both conducted in the US [86,87]. The study that assessed deaths from alcohol-related liver disease (*n* = 1) from a single liver transplant centre showed a higher number of deaths during the COVID-19’s declining phase compared with the previous year, but this difference was not statistically significant [86]. UK’s national statistics data found that there is overall an increasing trend of 1.6%. This trend increased even more rapidly and has been statistically higher during COVID-19 up to 4.6% after adjusting for age. This increase was evident in patients in the group 25–74 years of age [87].

### 3.5. Inequities

Some studies examined deaths of despair by population characteristics. Eighteen studies assessed the difference in sex and 14 in age groups for suicide death [24,30,32,38,47,48,49,51,52,53,54,55,57,60,61,63,88,89]. Studies conducted in Japan and Korea showed that the suicide rate significantly increased or showed higher rates among women and younger age groups than men and older age groups. Financial loss in these two groups was higher than other groups. In China, in addition to the younger age group, the elderly group’s suicide rate also increased while the overall trend of suicide was declining [63].

Two studies in the US assessed changes in the suicide rate among different ethnic groups, reporting an increase among Black and a decrease among White people. Occupations were measured in three studies in Japan, indicating that unemployed homemakers [52,53] and students had a higher risk of suicide [31,52,53]. However, no change was observed among those with recent unemployment in Australia [55]. A case series study also found a higher number of suicide deaths among daily wagers and self-employed compared with those who worked for the government during the pandemic [64]. The suicide rate in students showed no change during the school closure in Japan [50,52]. Studies investigating suicide motives (Japan and Australia; *n* = 2) and suicide methods (Japan and US, *n* = 2) found no differences.

Seven studies assessed changes in overdose-related death based on sex [65,72,73,75,78,82,87]. Of those, three reported a higher increase in overdose death in men than women [73,75,78]. Regarding the age group, three studies found no association [65,72,74], two observed a rise in younger people (less than 35 years of age [73] and 25 years of age [34]), and one reported an increase in adults older than 65 years of age [34]. A total of 2.6% increase in the average age of overdose deaths was also reported in one study [82]. One study also found a higher rise in overdose deaths among homeless individuals [72]. Among studies examining changes in overdose death based on ethnicity (all US; *n* = 5), four reported that either the increase was higher or it did not change in Black ethnicity, while it showed a decline in the White ethnicity.

## 4. Discussion

This review appraised evidence from 70 published studies concerning potential impacts of COVID-19 on deaths of despair. The rate of suicides was not observed to increase during the pandemic [32,38,45,55,57,90], though some studies indicated a potential drop in comparison with pre-pandemic years [26,27,28,33,46,54,58,60,61,63,89,91]. Only a few countries, such as Japan, reported contradictory results for suicide. Studies examining overdose death, however, mostly showed a higher rate of overdose death during the COVID-19 period compared with pre-pandemic years. Findings from the studies sub-analysis indicate that women, ethnic minorities, and younger age groups, may have suffered disproportionately more than other groups. Note that studies mainly conducted a preliminary data analysis, with several limitations, and the mid-to-longer-term impact of COVID-19 on deaths of despair has yet to fully emerge.

There were several limitations to the studies that may alter the results. Studies were either descriptive case studies or ecological or cross-sectional in design, mainly had low qualities, and a considerable number of them did not conduct any inferential analysis or only conducted a basic comparison without considering underlying confounders such as time trends and population on growth. This can cause a remarkable bias in findings. Data on most recent death cases, particularly in suicide and overdose cases, may be the least reliable and subject to undercounts, as unnatural death case examinations may take an extended amount of time. Further, during the pandemic, the data-collection processes may be disrupted further. Some of the studies were also published as editorials, such as a letter to an editor or commentary where they may not have always been subjected to an external peer-review process.

Findings regarding suicide death rates during the pandemic are consistent with a study that analysed data from 21 countries showing either no changes or reductions in suicide [8]. The lack of increase in suicides since the pandemic began can be attributed to various factors. Despite the early evidence highlighting that health measures such as lockdowns may heighten depression, anxiety, and suicidal thinking [1], country policies may have attenuated these adverse effects. Most of the studies have been conducted in high-income countries where welfare safety nets and, in particular, vaccination access, were often greater in comparison with low-income or lower-middle-income countries, which account for 75% [92] of the world’s suicides and might have been hit particularly hard by the pandemic. Note that some of these supports, such as financial aids, may now be reduced or halted [8]. For example, the observed initial support from the government has faded away over time in Austria [93] and Australia [56]. Thus, it is possible that the pandemic’s potential suicide-related effects are yet to occur even in countries with no current change. This is reflected in some subgroup analyses of the included studies indicating that disadvantageous groups showed a higher rate of suicide compared with the pre-pandemic period.

Drug overdose and drug-related liver disease deaths, on the other hand, seem to have increased or accelerated remarkably since the pandemic began, particularly in groups subject to inequity (some ethnic groups). Our findings regarding overdose deaths are consistent with a previous systematic review conducted on public health surveillance data published prior to September 2020 [5]. In addition, the current review also shows that this higher rise is mainly attributed to synthetic opioids, stimulants, and alcohol [82,94]. While these findings are preliminary and limited to a few counties (mostly US), they are concerning and call for urgent actions of policymakers to prevent drug-related deaths rooted in race equity. Strategies such as allowing longer prescription duration, mail, and remote supplying of medications to treat substance use disorders, providing safer drug alternatives such as tablet-based or low-release morphine have been suggested as new strategies to reduce harm and drug overdose [95].

Future studies may consider to examine factors such as environmental characteristics (e.g., nature) that may alleviate pandemic-related stress [96,97] and impacts on deaths of despair. In the current review, none of the included studies have investigated variation in deaths of despair regarding nature exposure during the pandemic. Enabling people to access natural settings on a more frequent basis can be a potential approach to alleviate health inequity through disruption of maladaptive rumination and social anxieties that sustain depression, loneliness and concomitant feelings of despair [98,99,100]. In addition, racial and gender disparity in society and the sense of loneliness can be alleviated by implementing a social psychology theory of superordinate goals to reduce conflicts and disconnection among social groups. Studies show superordinate goals, which refer to goals that require various social groups’ cooperation to accomplish, can create a sense of shared identity within communities and invert members’ perception of being from different groups into being from a single inclusive group [101,102].

## 5. Conclusions

This review highlights the need for more high-quality studies in general, and in low-middle income countries in particular, to identify the impact of COVID-19 on deaths of despair. Future studies may want to consider the contribution of personal, social, economic, and environmental factors that protect some groups while leaving others more vulnerable. Further, despite studies being at a preliminary stage, the change in overdose deaths is concerning and strategies are needed to prevent drug overdoses.

## Figures and Tables

**Table 1:** Inclusion and exclusion criteria of the review.

Component	Included	Excluded
Participants	▪ Healthy or unhealthy subjects of any ages or sex ▪ Human studies only	▪ Animal and lab-based studies
Intervention/issue	▪ COVID-19 period with or without (unlock) social isolation, stay-at-home order, quarantine, financial hardship and/or professional stressors	
Comparator	▪ Period prior to COVID-19 ▪ COVID-19 unlock period	
Outcomes	▪ Objective measures of deaths from suicide, poisoning, alcohol-related liver diseases or cirrhosis	▪ Subjective measures (e.g., self-report, media reports) ▪ Death due to other reasons such as suicide due to COVID-19 psychopathic or psychological effects, death due to criminal actions ▪ Non-fatal despair-related outcomes such as non-fatal suicide
Study design	▪ Peer-reviewed journal articles ▪ Editorials, case reports, case series ▪ Observational studies	▪ Conference abstracts and dissertations ▪ Reviews, qualitative studies, essays, opinion pieces ▪ Empirical studies without an outcome
Language	▪ English	▪ Any languages other than English

## Data Availability

All relevant data supporting the results and conclusion of this study is available in the main article and the Supplementary Materials.

## Supplementary Materials

The following supporting information can be downloaded at: https://www.mdpi.com/article/10.3390/ijerph191912835/s1, Table S1: Search strategy; Table S2: Study summary and characteristics for suicide outcome; Table S3: Study summary and characteristics for overdose death and drug-related liver disease death; Table S4: Quality assessment of included publications (*n* = 70).
