# Supplementary material for: Have Deaths of Despair Risen during the COVID-19 Pandemic? A Systematic Review
Source: Int J Environ Res Public Health. 2022 Oct 7;19(19):12835. doi: 10.3390/ijerph191912835 (PMC9564909; doi:10.3390/ijerph191912835)
Supplement: Supplementary file 1 [file ijerph-19-12835-s001.zip › ijerph-1870288-supplementary.pdf]

Supplementary materials

Table S1. Search strategy

EMBASE via Ovid (1947 to 2020) – 29 Aug 2021

Despair	1.	*hopelessness/
	2.	exp hopelessness/
	3.	Despair*.ti,ab,kw.
	4.	Hopeless*.ti,ab,kw.
	5.	defeat*.ti,ab,kw.
	6.	exp social defeat/
	7.	*guilt/
	8.	guilt*.ti,ab,kw.
	9.	worthless*.ti,ab,kw.
	10.	*pessimism/
	11.	pessimis*.ti,ab,kw.
	12.	((loss or lack or limited) adj3 hope).ti,ab,kw.
	13.	*anhedonia/
	14.	anhedonia*.ti,ab,kw.
	15.	*apathy/
	16.	apath*.ti,ab,kw.
	17.	self-harm.ti,ab,kw.
	18.	(drug* or alcohol).ti,ab,kw.
	19.	*depression/
	20.	*anxiety/
	21.	depress*.ti,ab,kw.
	22.	anxiet*.ti,ab,kw.
	23.	*sadness/
	24.	sadness*.ti,ab,kw.
	25.	*irritability/
	26.	*hostility/
	27.	(hostilit* or irritabilit*).ti,ab,kw.
	28.	*loneliness/
	29.	(loneliness or lonely).ti,ab,kw.
	30.	(Loss adj2 self).ti,ab,kw.
	31.	((inability or lack or loss) adj3 pleasure).ti,ab,kw.
	32.	(selfharm* or self-harm*).ti,ab,kw.
	33.	(opioid* or overdose* or heroin or opium or Fentan?l or methadone or methamphetamine or cocaine or substance abuse or substance misuse or substance addiction).ti,ab,kw.
	34.	(((economic or financial) adj1 (hardship* or stress* or problem* or cris?s or insecurit* or derpression*)) or recession* or austerity or fiscal cris?s or job loss* or banking cris?s or personnel downsi?ing or macroeconomic condition* or unemployment).ti,ab,kw.
	35.	((drug* or alcohol) adj1 (abuse or addiction or misuse or death* or disorder* or dependen*)).ti,ab,kw.

	36.	("drug use" or "alcohol use" or alcoholic* or alcoholism).ti,ab,kw.
Drug-related liver disease	37.	(alcoholic liver or toxic liver or chronic hepatitis or (fibrosis adj3 liver) or cirrhosis or poisoning*).ti,ab,kw.
	38.	*chronic hepatitis/ or alcohol liver cirrhosis/ or "substance use".ti,ab,kw.
	39.	1 or 2 or 3 or 4 or 5 or 6 or 7 or 8 or 9 or 10 or 11 or 12 or 13 or 14 or 15 or 16 or 17 or 19 or 20 or 21 or 22 or 23 or 24 or 25 or 26 or 27 or 28 or 29 or 30 or 31 or 32 or 33 or 34 or 35 or 36 or 37 or 38
Death	40.	*death/ or *fatality/
	41.	*mortality/
	42.	(fatal* or mortalit* or death*).ti,ab,kw.
Suicide	43.	*suicide/ or suicid*.ti,ab,kw.
	44.	(selfmutilat* or self-mutilat* or self?mutilation or automutilation or self?immolation or self?immolat* or self?inflict*).ti,ab,kw.
	45.	((kill* or hang*) adj1 self).ti,ab,kw.
	50.	40 or 41 or 42
	51.	39 and 50
	52.	51 or 42 or 43 or 44 or 45
	53.	limit 52 to (human and english language and covid-19)
	54.	limit 53 to conference abstracts
	55.	limit 53 to "systematic review"
	56.	53 not (54 or 55)

Table S2. Study summary and characteristics for suicide outcome

Author year	Country, region	Study Design	Population sample size %female Age	Pandemic start date	Pandemic time-period	Comparison time-period period/comparison	Background information of study pandemic period	Comparison	Direction (↑↓∅)
Anzai 2021 (41)	Japan, all	ES	General population<br>All<br>F: NA<br>Any age	Mar 2020	Mar - Jun 2020	Jan 2013 - Apr 2020	Lockdown	Before COVID-19 same time	M: ↓*<br>F: ↓ & then ↑
Barbic 2021 (42)	Canada, BC	ES	General population<br>All<br>F: NA<br>Any age	Mar 2020	Mar - Aug 2020	Jan 2010 - Mar 2020	First wave of pandemic, economical support provided by government	Period preceding COVID-19	↓
Behera 2021 (25)	India, two districts of New Delhi	ES	General population<br>All<br>F: NA<br>Any age	Mar 2020	25 Mar - 31May 2020	1 Jun - 31 Oct 2020	Lockdown	Pandemic post-lockdown period	↓* (Lockdown lower than post-lockdown)
					25 Mar - 31 May 2020	25 Mar - 31May 2019	Lockdown	Before COVID-19 same time	↓*
					1 Jun - 31 Oct 2020	1 Jun - 31 Oct 2019	Post-lockdown	Before COVID-19 same time	∅
Bray 2021 (26)	US, MD	Es	General population<br>All<br>F: NA<br>Any age	5 Mar 2020	5 Mar - 7 May 2020	5 Mar - 7 May, 2017 - 2019	Lockdown	Before COVID-19 same time	↓*
					8 May - 7 Jul 2020	8 May - 7 Jul, 2017 - 2019	Post-lockdown	Before COVID-19 same time	↓*
Calati 2021 (43)	Italy, Milan and Monza and Brianza	ES	Autopsy cases<br>All<br>F: NA<br>Any age	Mar 2020	T1: Jan - Apr 2020	CT1: Jan - Apr 2019	NR	Before COVID-19 same time	↓†
					Feb-21	Feb-19			↑
					Mar-21	Mar-19			↓†
					Apr-21	Apr-19			↑†
					2020	2019		Before COVID-19 same time	↓†

					2020	2016 - 2019		Before COVID-19 year by year comparison	↓†
Calderon-Anyosa 2021a [44]	Peru, all	ES	Adult population<br>All<br>F: NA<br>18 + y	Mar 2020	16 Mar - 30 Jun 2020	16 Mar - 30 Jun, 2018 - 2019	Lockdown	Before COVID-19 same time	↓(NR)
					1 Jul - 31 Dec 2020	1 Jul - 31 Dec, 2018 - 2019	Post-lockdown	Before COVID-19 same time	↓(NR)
Calderon-Anyosa 2021b [45]	Peru, all	ES	General population<br>All<br>F: NA<br>Any age	Mar 2020	16 Mar - Sep 2020	2017 - 1 Mar 2020	Mar - Jun lockdown Jun -forward easing of restrictions	Pre-lockdown annual slope, lockdown monthly difference, pre-lockdown slope interaction	M: ↓(NR); An increase in time trend slope (1.2 more deaths/million after lockdown compared to the pre-pandemic period<br><br>F: ↓(NR); no change in time trend slope
Deisenhammer 2021 [27]	Austria, state of Tyrol	ES	General population<br>All<br>F: NA<br>Any age	NR	1 Apr - 30 Sep 2020	2 Apr - 30 Sep, 2006 - 2019	NR (includes lockdown)	Before COVID-19 same time	↓*
					1 Apr - Jun 2020	1 Apr - Jun, 2006-2019	NR	Before COVID-19 same time	↓
					Jun - 30 Sep 2020	Jun - 30 Sep, 2006 - 2019	NR	Before COVID-19 same time	↓*
Dwyer 2021 [46]	Australia, VIC	ES	General population<br>All<br>F: NA<br>Any age	27 Feb 2020	Mar 2020 - Jan 2021	2015 - 2019	Lockdown and Post-lockdown periods	Before COVID-19 same time	↓*
Faust 2021b [47]	US, MA	ES	General population<br>All<br>F: NA<br>10 + y	Mar 2020	Mar - May 2020	Jan - May, 2015 - 2019	Lockdown	Before COVID-19 same time	∅
Faust 2021a [47]	US, all	ES	General population<br>All	Mar 2020	Mar - Aug 2020	Mar - Aug, 2015 - 2019	NR (includes lockdown)	Before COVID-19 same time	↓*

			F: NA<br>Any age						
Habu 2021 [48]	Japan, two cities of Okayama and Kibichuo	ES	General population<br>All<br>F: NA<br>Any age	Mar 2020	Mar - Aug 2020	Mar - Aug, 2018 - 2019	Lockdown and post-lockdown	Before COVID-19 same time	Ø
Isumi 2020 [49]	Japan, All	ES	Children<br>All<br>F: NA<br>< 20 y	Mar 2020	Mar - May 2020	Mar - May, 2018 - 2019	School closure	Before COVID-19 same time	Ø
Karakasi 2021 [29]	Greece, regional unit of Evros, Thrace	ES	General population<br>All<br>F: NA<br>Any age	Feb 2020	1 Mar - 15 May 2020	1 Mar - 15 May, 2010 - 2019	Lockdown	Before COVID-19 same time	Ø
Nomura 2021a and Nomura 2021b (update) [23, 24]	Japan, all	ES	General population<br>All<br>F: NA<br>Any age	Mar 2020	Mar – Dec 2020	Mar - Sep, 2016 - 2019	Lockdown (April-May)	Before COVID-19 same time (expected mortality)	M: Jan & Oct: 2-22% ↑*; Apr 4-14% ↓*<br><br>F: Feb & Apr: 1-18% ↓*; Jul-Dec (Post-lockdown): 21-85%↑*
Kim 2021b [30]	Korea, all	ES	General population<br>All<br>F: NA<br>Any age	Jan 2020	Jan - Aug 2020	Jan - Aug 2019	Mar highest number of COVID-19	Before COVID-19 same time	Total: ↓†<br><br>M: ↓<br><br>F: ↑
Leske 2021 [50]	Australia, QLD	ES	General population<br>All<br>F: NA<br>Any age	Mar - 2021 (restrictions), Feb covid arrival	Feb - Aug 2020	2015 - Jan 2020	Lockdown and Post-lockdown	Before COVID-19 same time	Ø
Marutani 2021 [31]	Japan, all	ES	University graduate students<br>All<br>F: 30<br>Any age	Apr 2020	Apr 2020 - Mar 2021	2002 - 2019	Courses all online	Before COVID-19 same time	↑†
Messina 2021 [32]	Italy, all	ES	Italian Police<br>All<br>F: NA<br>25 + y	Feb 2020	2020	2015-2019	Lockdown: Feb - Aug 2020	Before COVID-19 annual average	Ø

Mitchell 2021 [51]	US, CT	ES	General population<br>All<br>F: NA<br>Any age	Mar 2020	Mar - May 2020	Mar - May 2014-2019	Lockdown	Before COVID-19 same time	Lockdown vs 5 y average: 20% ↓†<br><br>Lockdown vs previous y: 13% ↓†
Ontiveros 2021§ [52]	US, CA	CS	Suicide cases reported to a poison centre<br>All<br>F: NA<br>Any age	Mar 2020	Mar - May 2020	Mar - May, 2018-2019	Lockdown	Before COVID-19 same time	↓
Osaki 2021 [53]	Japan, all	ES	General population<br>All<br>F: NA<br>Any age	Apr 2020	Mar - Dec 2020	Average of 3 y prior to covid	Lockdown and post-lockdown	Before COVID-19 (3 y average)	1st wave (Apr-May): overall: 18%↓*<br><br>2nd wave: F: ↑ *, M: ∅<br><br>3rd wave: overall: ↑*, F: 70% ↑*
Pirkis 2021 [8]	21 countries (16 high-income and 5 upper-middle-income)	ES	General population<br>All<br>F: NA<br>Any age	NR	1 Apr - 31 Jul 2020	Jan 2019 - Mar 2020	Lockdown and post-lockdown	Period preceding COVID-19	∅ or ↓
Pokhrel 2021 [37]	Nepal, all	ES	General population<br>All<br>F: NA<br>Any age	Mar 2020	Mar - Jun 2020	NR (pre-lockdown date not specified)	Lockdown	Before covid (period NR)	25% ↑†
Qin 2021 [38]	Norway, all	ES	General population<br>All<br>F: NA<br>Any age	Mar 2020	Mar - May 2020	Mar - May, 2014-2018	Lockdown	Before COVID-19 same time	∅
Radeloff 2021 [54]	Germany, Leipzig major city	ES	General population<br>All<br>F: NA<br>Any age	Mar 2020	T1: 17- 22 Mar 2020 & 6 Jun - 30 Sep 2020	CT1: Jan - 17 Mar 2020	T1: travel restriction (moderate)	Period preceding COVID-19	∅
					T2: 22 Mar - 5 Jun 2020	CT1: Jan - 17 Mar 2020	T2: travel, outing and socialising restriction (severe)	Period preceding COVID-19	T2 vs CT1: ↓*<br><br>T2 vs T1: ∅

					T overall:<br>Mar - Sep<br>2020	CT overall:<br>Mar - Sep<br>2010- 2019	Lockdown and post-lockdown	Before COVID-19 same time	∅
Sakamoto 2021 [55]	Japan, all	ES	General population<br>All<br>F: NA<br>Any age	Central and local Gov: Late Mar 20 20, National: Apr 2020	Apr - Nov 2020	Apr – Nov, 2016 - 2019	Lockdown (until May) and post-lockdown	Before COVID-19 same time	M: Oct - Nov: ↑*<br>F: Jul - Nov: ↑*
Sengupta 2020 [56]	India, Cooch Behar (4th most populous state)	CS	Autopsy cases<br>All (n=335)<br>F: NA<br>Any age	25 Mar 2020	25 Mar - 25 Apr 2020	Apr 2019<br>Jan 2020<br>Feb 2020<br>Mar 2020	Lockdown	Period preceding COVID-19 and before COVID-19 same time	↑ † regardless of comparison period
Seposo 2021 [36]	Japan, all	ES	General population<br>All<br>F: NA<br>Any age	Apr 2020	Apr - Dec 2020	Jan 2010 - Mar 2020	Lockdown: Apr - May 2020 and post-lockdown	Before COVID-19 (10 y average)	Relative risk change: ↑*<br>Crude comparison: ↓*
Shrestha 2021 [57]	Nepal, Kathmandu (Dhulikhel rural tertiary)	CS	Patients with self-harm at an emergency department<br>All<br>F: NA<br>Any age	24 Mar 2020	24 Mar - 23 Jun 2020	25 Mar-23 Jun 2019	Lockdown	Period preceding COVID-19	↑†
						24 Dec 2019 - 23 Mar 2020		Period preceding COVID-19	↑†
Tanaka 2021 [58]	Japan, all	ES	General population<br>All (126M)<br>F: NA<br>Any age	NR	Outbreak 1: Feb - Jun 2020	Nov 2016 - Feb 2020	1st Outbreak	Before COVID-19	14% ↓*
					outbreak 2: Jul - Oct 2020		2nd outbreak		16% ↑*
Ueda 2021 [59]	Japan, all	ES	General population<br>All<br>F: NA<br>Any age	NR	Apr - Oct 2020	2017- Feb 2019	Two outbreaks	Before COVID-19 trends (3 y)	Feb - Jun: ↓*<br>Jul - Oct: ↑*
Zheng 2021[60]	China, Guangdong	ES	General population<br>All	Jan 2020	1 Jan - 30 Jun 2020	1 Jan - 30 Jun 2019	Lockdown and post-lockdown	Before COVID-19 same time	Overall: (-)18.46% ↓*

			F: NA<br>Any age						0-14 y: 139.2% ↑*<br><br>70-79 y: 16.8% ↑*
Carlin 2021 [61]	Austria, Vienna	CS	A trauma centre admission cases<br>All<br>F: NA<br>Any age	16 Mar 2020	16 Mar - 15 May 2020	16 Mar - 15 May, 2015-2019 (except for 2017 as the hospital was closed)	Lockdown	Before COVID-19 same time	∅
Knipe 2021§ [33]	Sri Lanka,	CS	Patients presented with self-poisoning<br>All<br>F: NA<br>Any age	20 Mar 20	20 Mar - 31 Aug 2020	1 Jan 2019 - 19 Mar 2020	Lockdown and post-lockdown (lockdown lifted in 28 Jun 2020)	Period preceding COVID-19	↓†
Sakelliadis 2020 [62]	Greece, Athens	CS	Autopsy cases<br>All<br>F: NA<br>Any age	17 Mar 20	17 Mar - 15 Apr 2020	17 Mar - 15 Apr 2019	Lockdown	Before COVID-19 same time	∅
Kumar 2021‡ [63]	India, West Bengal	CS	Autopsy cases<br>All<br>F: NA<br>Any age	Mar 2020	Mar - Sep 2020	None	Lockdown	NA	27.8% and 17.9% of autopsy cases were related to hanging and poisoning, respectively.
Choudhury 2020 [64]	India, Lucknow	CSS	Suicide cases<br>All (n=59)<br>F: 44.4%<br>Any age	Mar-20	24 Mar-31May 2020	NA	Lockdown	NA	Economic factors accounted for 49% of cases, domestic conflicts 23.7%, psychological and emotional factors 27.1%.<br><br>64% of suicides were in the group of 18 -35 y.<br><br>The majority of suicides were done by hanging (93.2%).<br><br>The daily wagers and the self-employed citizens were most affected (36%).

Forouzanfar 2020 [65]	Iran, Tehran	CR	Family suicide A middle-aged mother and a 32 y son (n=2)	NR	NR	NA	NR	NA	The reason for suicide was the death of the father due to COVID-19<br><br>Method: poisoning (aluminium phosphide)<br><br>Background: wealthy and highly educated, high Socioeconomic status
Pirnia 2020 [66]	Iran, Tehran	CR	Family suicide A teenager son and 52 y mother (n=2)	NR	18-20 Mar 2020	NA	Lockdown	NA	Father died due to COVID-19 few weeks back. Lack of mourning rites specified as a reason for his depression symptoms and suicide.<br><br>Mother committed suicide by taking 2 aluminium phosphide pills two days after his son death.
Uğurlu 2020 [67]	Turkey	CR	Suicide case 34 y male	NR	02-Apr-20	NA	NR	NA	Reason: Mixed anxiety and depression induced by the COVID-19 pandemic stressor<br><br>Method: gun shot at his home.<br><br>Background: no know history of psychiatric or physiological disorder, no regular job

§ Suicide due to poisoning only, ‡ hanging and poisoning
† No inferential comparison
↑ denotes increase; ↓denotes decrease; ∅ no considerable change; * $p<0.05$
CS cross-sectional study; CT control time; CSS case series study; CR case-report study; ES ecological study; F female; M male, NA not applicable; T time

Table S3. Study summary and characteristics for overdose death and drug-related liver disease death

Author Year	Country, region	Study Design	Population sample size %female Age	Pandemic start date	Pandemic time-period	Comparison time-period period	Background information	Death outcome	Comparison	Direction (↑↓∅)
Appa 2021 [68]	US, San Francisco	ES	General population All F: NA Any age	Mar 2020	17 Mar - 30 Nov 2020	1 Jul 2019 - 16 Mar 2020	NR	Drug overdose (unintentional)	Period preceding COVID-19	↑*
					2020	2017 - 2019			Before COVID-19 (monthly average)	↑†
Brothers 2021 [69]	US, CT	CSS	Methadone recipients All F: NA Any age	16 Mar 2020	Apr - Aug 2020	Apr – Aug, 2015 - 2019	Methadone administration has relaxed	Methadone overdose	Before COVID-19 same time	∅ (no change compared to other opioids' trends)
DiGennaro 2021 [70]	US, MA	ES	General population All F: NA Any age	Mar 2020	24 Mar - 8 Nov 2020	24 Mar - 8 Nov 2019	Lockdown	Drug overdose (all intent)	Before COVID-19 same time	∅
						24 Mar - 8 Nov 2018				∅
Faust 2021a [28]	US, all	ES	General population All F: NA Any age	Mar 2020	Mar - Aug 2020	Mar - Aug, 2015 - 2019	NR (includes lockdown)	Drug overdose (all intent)	Before COVID-19 same time	↑*
Friedman 2021 [71]	US, all	ES	General population All F: NA Any age	Mar 2020	Jan - Jul 2020	Jan - Jul, 2015 - 2019	NR (includes lockdown)	Drug overdose (all intent)	Before COVID-19 same time	↑†
Aghababaeian 2020 [72]	Iran, All	ES	General population All F: NA Any age	Feb 2020	7 Mar - 8 Apr 2020	Mar - Apr 2019	An increase in the availability of illegal alcohol-based products	Alcohol (Methanol)	Before COVID-19 same time	↑†
						Apr 2016 - Sep 2018			Before COVID-19 overall time	↑†

									(annual average	
Karakasi 2021 (29)	Greece, regional unit of Evros, Thrace	ES	General population<br>All<br>F: NA<br>Any age	Feb 2020	1 Mar - 15 May 2020	1 Mar - 15 May 2010 - 2019	Lockdown	Unintentional poisoning	Before COVID-19 same time	↑*
Khatri 2021 (73)	US, Philadelphia	ES	General population<br>All<br>F: NA<br>Any age	Mar 2020	Mar - May 2020	Mar - May 2019	Lockdown	Opioid overdose (unintentional)	Before COVID-19 same time	Black: ↑*<br>Non-Hispanic White: ↓<br>Hispanic: ∅
						Dec 2019 - Feb 2020			Period preceding COVID-19	Black: ↑*<br>Non-Hispanic White: ∅<br>Hispanic: ∅
Kim 2021a (74)	US, all	ES	General population<br>All<br>F: NA<br>Any age	Mar 2020	Q1 - Q3 2020	Q1 - Q4, 2017 - 2019	NR	Chronic Liver Disease and Cirrhosis (combined)	Before COVID-19 same time	4.6% ↑* (age-adjusted)
Kitchen 2021 (75)	Canada, Ontario	ES	General population<br>All<br>F: NA<br>15 + y	15 Mar 2020	15 Mar - Sep 2020	15 Mar - Sep 2019	Lockdown	Opioid overdoses (all intent)	Period preceding COVID-19	135% ↑*
						Sep 2019 - 15 Mar 2020			Before COVID-19 same time	135% ↑*
Hassanian-Moghaddam 2020 (76)	Iran, all	ES	General population<br>All<br>F: NA<br>Any age	Feb 2020	Feb - May 2020	None (outbreak in Libya)	An increase in the availability of illegal alcohol	Alcohol (Methanol)	None (Second-largest methanol outbreak in history)	↑†
Mariottini 2021 (77)	Finland, all	CS	Autopsy cases<br>All<br>F: NA<br>Any age	Mar 2020	Jan - Aug 2020	2015 - 2019	lockdown and post-lockdown (Jun), reduced	3 most common drugs	Before COVID-19 same time	↑†

							access to harm-reduction services			
Mason 2021b (40)	US, Cook County	ES	General population<br>All<br>F: NA<br>Any age	Mar 2020	21 Mar - 30 May 2020	CT1: 15 Dec 2019 – 20 Mar 2020	Lockdown, interruptions and changes in the illicit drug supply	Opioid overdose (all intent)	Period preceding COVID-19 (average)	↑†
						CT2: 1 Jan 2018 - 14 Dec 2019				↑†
					31 May - 6 Oct 2020	CT1: 15 Dec 2019 – 20 Mar 2020	Post - lockdown		Period preceding COVID-19 (average)	↑†
						CT2: 1 Jan 2018 - 14 Dec 2019				∅
Mason 2021a (78)	US, Cook County	ES	General population<br>All<br>F: NA<br>Any age	Mar 2020	21 Mar - 30 May 2020	CT1: 15 Dec 2019 – 20 Mar 2020<br><br>CT2: 1 Jan 2018 - 14 Dec 2019	Lockdown, interruptions in the illicit drug supply and in-person service	Opioid overdose (all intent)	Period preceding COVID-19 (average)	Lockdown vs CT1 & CT2: ↑
					6 Jun - 23 Dec 2020		Post-lockdown, interruptions in the illicit drug supply and in-person service			Post-lockdown vs CT1 & CT2: ↑<br><br>Lockdown vs post-lockdown: ↑
Shokoohi 2020 (79)	Iran, all	CS	General population<br>All<br>F: NA<br>Any age	Feb 2020	Feb - Apr 2020	Feb - Apr 2019	An increase in the availability of illegal alcohol-based products	Alcohol (Methanol)	Before COVID-19 same time	↑†
						Mar 2016 - Aug 2018 (29 months)			Before COVID-19 (29 months)	↑†

Patel 2021 [80]	Us, Birmingham	CS	Patients admitted for an overdose<br>One hospital's patients<br>F: NR<br>Any age	NR	1 Jan - 31 Oct 2020	1 Jan - 31 Oct, 2019	Lockdown and post-lockdown	Opioid overdose (all intent)	Before COVID-19 same time	Overall ↓†<br><br>Black ~20%↑†<br><br>White ~30%↓†
Rutledge 2021 [81]	US, NY	CS	Alcohol-associated liver disease (ALD) patients<br>All<br>ALD patients referred to a liver transplantation centre<br>F: 37-50%<br>Any age	Mar 2020	T2: 23 Apr -23 Aug 2020	1 Jan - 21 Mar 2020	Post-lockdown	ALD	Period preceding COVID-19	↑
Shreffler 2021 [82]	US, KY (Jefferson County, most populated)	CS	General population<br>All<br>F: NA<br>Any age	6 Mar 2020	6 Mar - 25 Jun 2019	6 Mar - 25 Jun 2019	Lockdown	Drug overdose (all intent)	Before COVID-19 same time	↑†
						15 Nov 2019 - 5 Mar 2020			Period preceding COVID-19	↑†
Vieson 2021 [83]	US, OH	ES	Adult population<br>All<br>F: NA<br>18 + y	NR	Apr - Jun 2020	T1: Expected value for 2020	NR	Opioid overdose (all intent)	Before COVID-19, expected mortality	↑*
						T2: last peak Q4 2017				null
Rodda 2020 [84]	US, San Francisco	ES	General population<br>All<br>F: NA<br>15 + y,<br>Median: 54 y (IQR: 35-56)	15 Mar 2020	16 Mar - 18 Apr, 2020	1 Jan - 15 Mar 2020	Lockdown	Opioid overdose (accidental)	Period preceding COVID-19 and before COVID-19 same time	↑†
						16 Mar - 18 Apr, 2018-2019				↑†

Yazdi-Feyzabadi 2021 [35]	Iran, 13 provinces	ES	General population<br>All<br>F: NA	Feb 2020	Apr-20	None	An increase in the availability of illegal alcohol-based products	Alcohol (Methanol)	None	A high number of poisonings
Zhang 2021 [34]	US, OH	ES	General population<br>All<br>F: NA<br>Any age	15 Mar 2020	Mar - 10 Oct 2020	1 Jan 2018 - 15 Mar 2020		Drug Overdose (all intent)	Period preceding COVID-19	Up to 76.8% ↑† then dropped to an earlier rate when unemployment reduced.
Zheng 2021 [60]	China, Guangdong	ES	General population<br>All<br>F: NA<br>Any age	Jan 2020	1 Jan - 30 Jun 2020	1 Jan - 30 Jun 2019	Lockdown and post-lockdown	Poisoning (accidental)	Before COVID-19 same time	↓†
Pines 2021 [85]	US, 18 states	ES	Overdose visits to EDs<br>All EDs<br>F: NA<br>Any age	13 Mar 2021	13 Mar - 31 Jul 2020	14 Mar- 31 Jul 2019	NR	Drug overdose death (ED deaths or deaths on arrival, all intent)	Before COVID-19 same time	Total: 2.51 times ↑†<br>Opioid: 2.5 times ↑†<br>Alcohol:5.25 times ↑†<br>Other drugs: 2 times ↑†
Slavova 2021 [86]	US, KY	ES	Kentucky State Ambulance cases<br>All<br>F: NA<br>Any age	5 Mar 2020	6 Mar - 26 Apr 2020	January 14, 2020, to March 5, 2020	Lockdown	Opioid overdose death at the scene (all intent)	Period preceding COVID-19	↑†
Glober 2020 [87]	US, Marion county	ES	General population<br>All<br>F: NA	25 Mar 2020	25 Mar - 7 July 2020	26 Mar -7 July 2019	Lockdown and post-lockdown (early May)	Any drug overdose (including suspected cases)	Before COVID-19 same time	↑*
						1 Jan 2019 - 24 Mar 2020			Period preceding COVID-19	↑*

Congdon 2021 [88]	UK, London	CS	Patients registered at two substance misuse services<br>All<br>F: NA<br>Any age	Mar 2020	Mar - Apr 2020	Mar - Apr 2019	Reduced face-to-face interactions in the service	Drug overdose (accidental)	Before COVID-19 same time	∅
UK National Statistical Bulletin 2021 [89]	UK, England, and Wales	ES	General population<br>All<br>F: NA	NR	Q1 - Q4 2020	2001-2019	lockdown and post-lockdown	Alcohol	Before COVID-19 same time	2020 vs 2019: 19.6% ↑*<br><br>Q2 - Q4 2020 vs 2001 - Q1 2020: 17-28% ↑*<br><br>Continuous since Q2: ↑* trend<br><br>2020 Q1 2020 vs 5 y average: ∅
Yip 2020 [39]	US, Arizona, and New Mexico	CSS	Methanol poisoning cases<br>All<br>F: NA<br>Any age	NR	1 May - 30 Jun 2020	NA	NR	Alcohol (Methanol)	None	4 out of 15 poisoning cases were related to methanol
Simani 2020 [90]	Iran, Tehran	CSS	Methanol poisoning cases underwent computed tomography scan<br>n=40<br>F: 23% (non-survival)<br>Age: 42.4 ± 14.0 y (non-survival)	NR	Mar - Apr 2020	NA	NR	Alcohol (Methanol)	None	Mortality rate: 55% (n=22)<br><br>The lower mortality rate in cases with chronic alcohol consumption than those who drank alcohol for the first time ($p<0.05$).<br><br>No significant difference in the blood level of Amphetamine, history of other illegal drugs, or COVID-19 infection was found

										between survivals and non-survivals.<br><br>Non-survival characteristics:<br>Alcohol history: 18 (81.8%)<br>Amphetamine level: 6 (27.3%)<br>Illicit drug history: 10 (45.5%)
Dumollard 2021 [91]	France	CR	Death case due to Isopropyl Alcohol poisoning (n=1)<br>F: 0%<br>33 y	NR	NR	NA	NR	Alcohol (isopropyl and acetone) (cleaning solvent)	NA	Found dead in his home with a bottle of isopropyl alcohol liquid found close to him<br><br>Background: history of drug addiction, psychosis, and an attempt of hanging himself.

† No inferential comparison
↑ denotes increase; ↓denotes decrease; ∅ no considerable change; * p<0.05
ALD Alcohol-associated liver disease; CS cross-sectional study; CT control time; ED emergency department; ES ecological study; F female; IQR interquartile range; M male, NA not applicable; NR not reported; Q quartile; T time

Table S4. Quality assessment of included publications (n=70)

a) Cross-sectional studies

For the list of questions assessed for each type of studies, see https://www.nhlbi.nih.gov/health-topics/study-quality-assessment-tools

For ecological studies, the adapted questions are:

Q14 (modified): Were key potential confounding variables measured at the ecological unit level and adjusted statistically for their impact on the relationship between exposure(s) and outcome(s)?

Q15 (added): Was spatial autocorrelation addressed?

Q16 (added): Was variation of outcome distribution within each unit of analysis accounted for in any way?

Author Year	Q1	Q2	Q3	Q4	Q5	Q6	Q7	Q8	Q9	Q10	Q11	Q12	Q13	Q14	Q15	Q16	Total score	Total applicable	Ratio
Anzai 2021	Y	Y	Y	N	NA	N	N	N	Y	N	Y	Y	NA	Y	N	N	7	14	0.50
Appa 2021	Y	Y	Y	N	NA	N	N	N	Y	N	Y	Y	NA	N	N	N	6	14	0.43
Barbic 2021	Y	Y	Y	N	NA	N	N	N	Y	N	Y	Y	NA	Y	Y	N	8	14	0.57
Behera 2021	Y	Y	Y	N	NA	N	N	N	Y	N	Y	Y	NA	N	N	N	6	14	0.43
Bray 2021	Y	Y	Y	N	NA	N	N	Y	Y	Y	Y	Y	NA	N	N	N	8	14	0.57
Brothers 2021	Y	Y	Y	N	NR	N	N	N	Y	N	Y	Y	NA	N	NA	NA	6	13	0.46
Calati 2021	Y	Y	Y	N	NA	N	N	N	N	N	Y	Y	NA	N	N	N	5	14	0.36
Calderon-Anyosa 2021a	Y	Y	Y	N	NA	N	N	Y	Y	Y	Y	Y	NA	N	NR	NR	8	14	0.57
Calderon-Anyosa 2021b	Y	Y	Y	N	NA	N	N	Y	Y	Y	Y	Y	NA	Y	Y	Y	11	14	0.79
Deisenhammer 2021	Y	Y	Y	N	NA	N	N	Y	Y	Y	Y	Y	NA	Y	NR	NR	9	14	0.64
DiGennaro 2021	Y	Y	Y	N	NA	N	N	N	Y	N	Y	Y	NA	N	NR	NR	6	14	0.43
Dwyer 2021	Y	Y	Y	N	NA	N	N	N	Y	N	Y	Y	NA	Y	Y	NA	8	13	0.62
Faust 2021b	Y	Y	Y	N	NA	N	N	N	Y	N	Y	Y	NA	Y	Y	NR	8	14	0.57
Faust 2021a	Y	Y	Y	N	NA	N	N	N	Y	N	Y	Y	NA	Y	Y	NR	8	14	0.57
Friedman 2021	Y		Y	N	NA	N	N	N	N	N	Y	Y	NA	N	N	N	4	14	0.29
Habu 2021	Y	Y	Y	N	NA	N	N	N	Y	N	N	Y	NA	N	NR	NR	5	14	0.36
Hamdanieh 2020	Y	Y	Y	N	NA	N	N	N	Y	N	Y	Y	NA	N	NR	NR	6	14	0.43
Isumi 2020	Y	Y	Y	N	NA	N	N	N	Y	N	Y	Y	NA	N	NR	NR	6	14	0.43
Karakasi 2021	Y	Y	Y	N	NA	N	N	N	Y	N	NR	Y	NA	N	NR	NR	5	14	0.36
Nomura 2021a	Y	Y	Y	N	NA	N	N	N	Y	N	Y	Y	NA	Y	NR	NR	7	14	0.50
Nomura 2021b	Y	Y	Y	N	NA	N	N	N	Y	N	Y	Y	NA	Y	NR	NR	7	14	0.50
Khatri 2021	Y	Y	Y	N	NA	N	N	N	Y	N	Y	Y	NA	N	N	N	6	14	0.43
Kim 2021b	Y	Y	Y	N	NA	N	N	N	Y	N	Y	Y	NA	N	N	N	6	14	0.43
Kim 2021a	Y	Y	Y	N	NA	N	N	N	N	N	Y	Y	NA	Y	N	N	6	14	0.43
Kitchen 2021	Y	Y	Y	N	NA	N	N	N	Y	N	Y	Y	NA	N	N	N	6	14	0.43
Hassanian-Moghaddam 2020	Y	Y	Y	N	NA	N	N	N	Y	N	Y	Y	NA	N	N	N	6	14	0.43
Kumar 2021	Y	Y	Y	Y	NA	N	N	N	Y	N	Y	Y	NA	N	N	N	7	14	0.50
Leske 2021	Y	Y	Y	N	NA	N	N	N	Y	N	Y	Y	NA	N	Y	NR	7	14	0.50
Mariottini 2021	Y	Y	Y	Y	NA	N	N	N	N	N	Y	Y	NA	N	N	N	6	14	0.43
Marutani 2021	Y	Y	Y	N	NR	N	N	N	Y	N	N	NR	Y	N	N	N	5	16	0.31
Mason 2021b	Y	Y	Y	N	NA	N	N	N	Y	N	Y	Y	NA	N	N	N	6	14	0.43
Mason 2021a	Y	Y	Y	N	NA	N	N	Y	Y	Y	Y	Y	NA	N	N	N	8	14	0.57

Messina 2021	Y	Y	Y	N	NA	N	N	N	N	N	NR	Y	NA	N	N	N	4	14	0.29
Mitchell 2021	Y	Y	Y	N	NA	N	N	N	Y	N	Y	Y	NA	N	N	N	6	14	0.43
Shokohi 2020	Y	Y	Y	N	NA	N	N	N	Y	N	Y	Y	NA	N	N	N	6	14	0.43
Ontiveros 2021	Y	Y	Y	N	NA	N	N	N	Y	N	N	Y	NA	N	N	N	5	14	0.36
Osaki 2021	Y	Y	Y	N	NA	N	N	N	Y	N	Y	Y	NA	N	N	N	6	14	0.43
Patel 2021	Y	Y	Y	N	NA	N	N	N	NR	N	Y	Y	NA	N	NR	NR	5	14	0.36
Pirkis 2021	Y	Y	Y	N	NA	N	N	N	Y	N	Y	Y	NA	Y	NR	NR	7	14	0.50
Pokhrel 2021	Y	Y	Y	N	NA	N	N	N	Y	N	Y	Y	NA	N	NR	NR	6	14	0.43
Qin 2021	Y	Y	Y	N	NA	N	N	N	Y	N	Y	Y	NA	N	NR	NR	6	14	0.43
Radeloff 2021	Y	Y	Y	N	NA	N	N	Y	Y	Y	Y	Y	NA	N	NR	NR	8	14	0.57
Rutledge 2021	Y	Y	Y	Y	NA	Y	N	Y	Y	Y	Y	Y	Y	N	NR	NR	12	15	0.80
Sakamoto 2021	Y	Y	Y	N	NA	N	N	N	Y	N	Y	Y	NA	N	NR	NR	6	14	0.43
Sengupta 2020	Y	Y	Y	Y	NR	N	N	N	Y	N	Y	Y	NA	N	N	N	7	15	0.47
Seposo 2021	Y	Y	Y	N	NA	N	N	N	Y	N	Y	Y	NA	Y	NR	NR	7	14	0.50
Shreffler 2021	Y	Y	Y	Y	NR	N	N	N	Y	N	Y	Y	NA	N	NR	NR	7	15	0.47
Shrestha 2021	Y	Y	Y	Y	NR	N	N	N	Y	N	Y	Y	NA	N	N	N	7	15	0.47
Tanaka 2021	Y	Y	Y	N	NA	N	N	Y	Y	Y	Y	Y	NA	Y	NR	NR	9	13	0.69
Ueda 2021	Y	Y	Y	N	NA	N	N	N	Y	Y	Y	Y	NA	Y	NR	NR	8	13	0.62
Vieson 2021	Y	Y	Y	N	NA	N	N	N	Y	Y	Y	Y	NA	Y	NR	NR	8	13	0.62
Rodda 2020	Y	Y	Y	N	NA	N	N	N	Y	N	Y	Y	NA	N	N	N	6	14	0.43
Yazdi-Feyzabadi 2021	Y	Y	Y	N	NA	N	N	N	Y	N	Y	Y	NA	N	N	N	6	14	0.43
Zhang 2021	Y	Y	Y	N	NA	N	N	N	Y	N	Y	Y	NA	N	N	N	6	14	0.43
Zheng 2021	Y	Y	Y	N	NA	N	N	N	Y	N	Y	Y	NA	N	N	N	6	14	0.43
Pines 2021	Y	Y	Y	N	NA	N	N	N	Y	N	Y	Y	NA	N	N	N	6	14	0.43
Carlin 2021	Y	Y	Y	Y	NR	N	N	N	Y	N	Y	Y	NA	N	NA	NA	7	13	0.54
Slavova 2021	Y	Y	Y	N	NA	N	N	N	Y	N	Y	Y	NA	N	N	N	6	14	0.43
Glober 2020	Y	Y	Y	N	NA	N	N	N	Y	N	Y	Y	NA	Y	Y	N	8	14	0.57
Congdon 2021	Y	Y	Y	Y	NA	N	N	N	Y	N	Y	Y	NA	N	NA	NA	7	12	0.58
UK National Statistical Bulletin 2021	Y	Y	Y	N	NA	N	N	N	N	N	Y	Y	NA	N	NR	NR	5	14	0.36
Knipe 2021	Y	Y	Y	Y	NR		N	N	Y	N	Y	Y	NA	N	NA	NA	7	13	0.54
Sakelliadis 2020	Y	Y	Y	Y	NR		N	N	Y	N	Y	Y	NA	N	NA	NA	7	13	0.54

N, no; NA, not applicable; NR, not reported; Y, Yes

b) Case series

For the list of questions assessed for each type of studies, see https://jbi-global-wiki.refined.site/space/MANUAL/3290006117/Appendix+7.3+Critical+appraisal+checklists+for+case+series

Author Year	Q1	Q2	Q3	Q4	Q5	Q6	Q7	Q8	Q9	Q10	Total score	Total applicable	Ratio
Choudhury 2020	Y	Y	Y	Y	Y	Y	Y	NA	Y	N	8	9	0.89
Yip 2020	Y	N	Y	Y	Y	N	Y	NA	Y	NA	6	8	0.75
Simani 2020	Y	Y	N	Y	Y	Y	Y	NA	Y	Y	8	9	0.89

N, no; NA, not applicable; NR, not reported; Y, Yes

c) Case reports

For the list of questions assessed for each type of studies, see https://jbi-global-wiki.refined.site/space/MANUAL/3290006119/Appendix+7.4+Critical+appraisal+checklist+for+case+reports

Author Year	Q1	Q2	Q3	Q4	Q5	Q6	Q7	Q8	Total score	Total applicable	Ratio
Forouzanfar 2020	Y	N	Y	Y	NA	NA	NA	Y	4	5	0.80
Pirnia 2020	Y	N	N	N	NA	NA	NA	Y	2	5	0.40
Uğurlu 2020	Y	Y	Y	Y	NA	NA	NA	Y	5	5	1.00
Dumollard 2021	Y	N	Y	Y	NA	NA	NA	Y	4	5	0.80

N, no; NA, not applicable; NR, not reported; Y, Yes
